# Preclinical evaluation of the antipsychotic potential of the mGlu2-positive allosteric modulator JNJ-40411813

**DOI:** 10.1002/prp2.97

**Published:** 2015-01-30

**Authors:** Hilde Lavreysen, Xavier Langlois, Luc Ver Donck, José María Cid Nuñez, Stefan Pype, Robert Lütjens, Anton Megens

**Affiliations:** 1 Janssen Research & Development, Janssen Pharmaceutica NVBeerse, Belgium; 2 Janssen Research & Development, Janssen-CilagToledo, Spain; 3 Addex TherapeuticsGeneva, Switzerland

**Keywords:** 5HT_2A_ receptor antagonist, antipsychotic-like activity, JNJ-40411813, JNJ-42153605, LY404039, mGlu2, PAM, ritanserin

## Abstract

JNJ-40411813/ADX71149 (1-butyl-3-chloro-4-(4-phenylpiperidin-1-yl) pyridin-2(1H)-one) is a positive allosteric modulator (PAM) of the mGlu2 receptor, which also displays 5-Hydroxytryptamine (5HT_2A_) antagonism after administration in rodents due to a rodent-specific metabolite. JNJ-40411813 was compared with the orthosteric mGlu2/3 agonist LY404039 (4-amino-2-thiabicyclo [3.1.0] hexane-4,6-dicarboxylic acid 2,2-dioxide), the selective mGlu2 PAM JNJ-42153605 (3-(cyclopropylmethyl)-7-(4-phenylpiperidin-1-yl)-8-(trifluoromethyl)[1,2,4]triazolo[4,3-a]pyridine) and the 5HT_2A_ antagonist ritanserin in rodent models for antipsychotic activity and potential side effects, attempting to differentiate between the various compounds and mechanisms of action. In mice, JNJ-40411813, JNJ-42153605, and LY404039 inhibited spontaneous locomotion and phencyclidine- and scopolamine-induced but not d-amphetamine-induced hyperlocomotion; the 5HT_2A_ antagonist ritanserin inhibited only spontaneous locomotion and phencyclidine-induced hyperlocomotion. As measured by 2-deoxyglucose uptake, all compounds reversed memantine-induced brain activation in mice. The two mGlu2 PAMs and LY404039, but not ritanserin, inhibited conditioned avoidance behavior in rats. Like ritanserin, the mGlu2 ligands antagonized 2,5-dimethoxy-4-methylamphetamine-induced head twitches in rats. LY404039 but not the mGlu2 PAMs impaired rotarod performance in rats and increased the acoustic startle response in mice. Our results show that although 5HT_2A_ antagonism has effect in some models, mGlu2 receptor activation is sufficient for activity in several animal models of antipsychotic activity. The mGlu2 PAMs mimicked the in vivo pharmacodynamic effects observed with LY404039 except for effects on the rotarod and acoustic startle, suggesting that they produce a primary activity profile similar to that of the mGlu2/3 receptor agonist while they can be differentiated based on their secondary activity profile. The results are discussed in light of clinical data available for some of these molecules, in particular JNJ-40411813.

## Introduction

The metabotropic glutamate 2 (mGlu2) receptor is a G protein-coupled autoreceptor present on glutamatergic nerve terminals. mGlu2 receptor activation reduces excitatory transmission and has emerged as a viable target for treating pathological conditions that are characterized by persistent glutamatergic dysfunction, such as schizophrenia (Marek et al. 2010).

The glutamate hypothesis of schizophrenia suggests that *N*-methyl-d-aspartate (NMDA) receptor hypofunction on GABAergic interneurons leads to disinhibition of excitatory projections to the prefrontal cortex, resulting in hyper-glutamatergic neurotransmission (Conn and Jones 2009). The fact that the NMDA antagonist phencyclidine (PCP) produces schizophrenia-like symptoms in healthy subjects and worsens psychosis in patients with schizophrenia (Jentsch and Roth 1999) further supports this hypothesis. The paradoxical increase in extracellular glutamate levels resulting from NMDA receptor hypofunction is confirmed by magnetic resonance spectroscopy studies in schizophrenic patients (Theberge et al. 2002). In stable schizophrenics treated with antipsychotics, however, a relative glutamate hypofunction in the left anterior cingulate cortex was noted (Theberge et al. 2003). Indeed, recent meta-analysis of ^1^H-MRS studies (Marsman et al. 2013) revealed that in patients with schizophrenia, glutamate concentrations decreased at a faster rate with age than in healthy controls. Hence, it is not yet completely understood how glutamatergic systems in schizophrenia are altered. These alterations may depend on the disease stage (acute vs. stable) as well as the nature of the symptoms.

In animals, mGlu2/3 receptor agonists have been shown to block PCP-induced increases in prefrontal glutamate levels and to reduce PCP-evoked hyperlocomotion (Moghaddam and Adams 1998; Cartmell and Schoepp 2000; Imre 2007). Also in other models of antipsychotic activity, such as conditioned avoidance behavior, mGlu2/3 receptor agonists are active (Rorick-Kehn et al. 2006; Megens et al. 2014).

Through the use of knockout mice lacking mGlu2 or mGlu3 receptors, mGlu2 rather than mGlu3 receptors seem responsible for the antipsychotic-like properties of mGlu2/3 receptor agonists (Fell et al. 2008; Woolley et al. 2008). Based upon the collective data obtained using mGlu2/3 selective agonists and mGlu2 or mGlu3 knockout mice, the mGlu2 receptor appears to be an interesting therapeutic target for the treatment of schizophrenia.

While initial clinical studies held promise for the use of mGlu2/3 receptor agonists-like LY404039 for the treatment of schizophrenia (Patil et al. 2007; Weinberger 2007), recent clinical trials demonstrated negative results, and more work is needed to understand the treatment potential of mGlu2(/3) receptor ligands (Kinon et al. 2011; Stauffer et al. 2013). Positive allosteric modulators (PAMs) of mGlu2 receptors, which bind to a site distinct from the glutamate-binding site (Conn and Jones 2009), may offer an alternative to nonselective mGlu2/3 receptor agonists. They can improve mGlu2 receptor selectivity and since they have minimal effect on their own but rather increase glutamate-induced signaling, reduce the risk of receptor over-activation and development of tolerance with repeated dosing (Galici et al. 2005; Fell et al. 2008; Woolley et al. 2008; Trabanco et al. 2011). Modulation of the mGlu2 receptor via PAMs showed anxiolytic- and antipsychotic-like effects in various preclinical studies (Galici et al. 2005, 2006; Johnson et al. 2005; Trabanco et al. 2011).

JNJ-40411813 (1-butyl-3-chloro-4-(4-phenyl-1-piperidinyl)-2 (1*H*)-pyridinone) is a novel mGlu2 receptor PAM (Lavreysen et al submitted[Bibr b47]); in vitro, it has a potency of approximately 150 nmol/L and an efficacy or *E*_max_ of about 270% at the human mGlu2 receptor. In vivo, JNJ-40411813 not only binds to the mGlu2 receptor (as demonstrated via ex vivo mGlu2 receptor occupancy), but also elicits functional mGlu2-mediated effects (as shown via sleep-wake EEG monitoring). In vitro, in addition to modulating mGlu2 receptors, JNJ-40411813 acts as a weak 5-Hydroxytryptamine (5HT_2A_) antagonist (IC_50_ = 708 nmol/L; *K*_b_ ∼ 1.1 *μ*mol/L). This 5HT_2A_ antagonist activity is more pronounced in vivo due to the generation of a rodent-specific metabolite (5HT_2A_ IC_50_ = 102 nmol/L). The combined mGlu2 PAM and 5HT_2A_ antagonistic properties of JNJ-40411813 in rodents offered the opportunity to test the in vivo effects of this unique combined pharmacological profile. In an attempt to differentiate between various compounds and mechanisms of action, we compared the effects of JNJ-40411813, the mGlu2/3 receptor agonist LY404039, the selective mGlu2 PAM JNJ-42153605 (Cid et al. 2012) and the 5HT_2A_ receptor antagonist ritanserin in animal models for antipsychotic activity and side effects. Since the preclinical observations with JNJ-40411813 have led to its selection for clinical studies, the results are also discussed in light of clinical data available for this molecule.

## Materials and Methods

### Study drugs

JNJ-40411813 (free base), JNJ-42153605 (free base), scopolamine (hydrobromide trihydrate), 2, 5 dimethoxy-4-methylamphetamine (hydrochloride; DOM) and phencyclidine (hydrochloride; PCP) were synthesized at Janssen Research & Development, Beerse, Belgium. LY404039 was synthesized at Janssen Research & Development or obtained from Sequoia Research Products. Memantine was purchased from Sequoia Research Products, Pangbourne, UK, and d-amphetamine (hemisulfate) from Certa, Braine-l’Alleud, Belgium.

JNJ-40411813 and JNJ-42153605 were dissolved in 10% or 20% hydroxypropyl-*β*-cyclodextrin containing 1 equivalent of hydrochloric acid and LY404039 was dissolved in saline containing 1 equivalent of sodium hydroxide; all other compounds were dissolved in saline. The respective vehicle solutions were administered as controls in each study. The solutions were stored at room temperature and protected from light.

All doses have been expressed in mg equivalents free base per kg body weight. All study drugs were administered in a volume of 10 mL/kg.

### Animals

The following strains of male mice were used: C57BL6J (C57BL/6N:Crlf) for the memantine-induced brain activation test; and NMRI [Crl:NMRI(Han)] for the locomotion and acoustic startle tests. Male Wistar (Crl:WI) rats were used for the DOM-induced head twitches and the conditioned avoidance test; and male Sprague–Dawley (Hsd:SD) rats were used for the rotarod test. The mice ranged in body weight between 20 and 40 g, whereas the rats ranged in body weight from 175 to 320 g. All animals were obtained from Charles River Breeding Laboratories (Sulzfeld, Germany or Chatillon-sur-Chalaronne, France) except the Sprague–Dawley rats which were obtained from Harlan, The Netherlands. The animals were group-housed under standard laboratory conditions (21 ± 2°C; 45–65% relative humidity; and 12/12 h light-dark cycle) and were acclimatized to the environment for at least 5 days before testing. All animals were fasted overnight prior to the start of the experiments (water ad libitum), except for the acoustic startle and rotarod tests (fed ad libitum). All experiments were carried out in strict accordance with Belgian Law (Royal decree on the protection of laboratory animals, 6 April 2010) and were approved by the ethical committee of Janssen Research and Development, LLC.

### Locomotor activity in mice

After arrival in the experimental room, mice were housed individually and allowed to acclimatize for at least half an hour before the start of the experiments. Although the studies were conducted during the light cycle (from 8:00 to 16:00 h), the procedure room was only sparsely lit (3–30 lx) to provide better contrast for the video tracking. Locomotion was measured in open field arenas (gray polyvinyl chloride **[**PVC] cylinders (height: 40 cm; diameter: 22.5 cm) placed on an infrared (8 × 8 light-emitting diode [LEDs]) lit box (white PVC; 40 × 40 × 12.5 cm^3^). An infrared-sensitive tube camera and a white light source (in arena: 4–7 lx) were mounted above the observation chamber to determine distance traveled using the Noldus Ethovision XT Video Tracking System (Version 3.1; Noldus, Wageningen, The Netherlands).

#### Spontaneous locomotion

Locomotion was measured over a 30-min period by introducing the mouse into the motor activity arena 30 min after pretreatment (s.c.) with test compounds (JNJ-40411813, JNJ-42153605, LY404039, ritanserin) or vehicle.

#### Hyperlocomotion induced by PCP, d-amphetamine, and scopolamine

**Figure 1:**
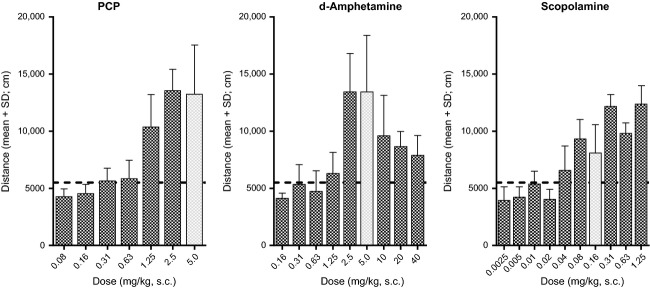
Dose–response relations for the locomotor stimulant effects of PCP, d-amphetamine, and scopolamine (*n* = 5–35 per dose group). The doses of the stimulants selected for the reversal studies have been highlighted.

Locomotion was measured over a 30-min period by introducing the mouse into the motor activity arena 30 min after pretreatment with test compound(s) or vehicle and simultaneous challenge with a stimulant agent (PCP [5.0 mg/kg, s.c.], d–amphetamine [5.0 mg/kg, s.c.] or scopolamine [0.16 mg/kg, s.c.]). The doses of the stimulant agents, selected based on the dose–response data in Figure1, reliably (≥95% responders) induced hyperlocomotion (>5500 cm). Table1 gives an overview of the averaged distance traveled in vehicle-pretreated control mice in each of the four locomotor activity assays, together with the percentages of false positives that responded to the criteria for inhibition of hyperlocomotion (<5500 cm) and blockade of locomotion (<2500 cm). ED_50_ values and the corresponding 95% confidence intervals were determined as described below both for inhibition of hyperlocomotion (<5500 cm) and for blockade of locomotion (<2500 cm).

**Table 1:** Distance traveled by vehicle-treated control mice.

	Group size	Distance traveled	False positives (%)	
Challenge (−0.5 h)	(*n*)	Mean ± SD (cm)	Inhibition <5500 cm	Blockade <2500 cm
None (spontaneous)	1074	4143 ± 947	89	4.1
PCP (5.0 mg/kg, s.c.)	2334	11,096 ± 3612	4.9	0.6
Scopolamine (0.16 mg/kg, s.c.)	1879	9944 ± 2010	2.0	0.3
d-Amphetamine (5.0 mg/kg, s.c.)	464	12,866 ± 4445	2.8	0.0

Mice were either not challenged (spontaneous locomotion) or treated with PCP, scopolamine or d-Amphetamine (0.5 h pretreatment interval). The percentages of false positives responding to the criteria for inhibition (<5500 cm) and blockade (<2500 cm) in the control population are also listed. PCP, phencyclidine.

In a separate study, the effect of JNJ–40411813 (10 mg/kg, s.c., 30 min) versus vehicle (10 mL/kg, s.c.; 30 min) on the dose–response curve for the locomotor stimulant effect of PCP (0, 0.63, 1.25, 2.5, 5.0, 10 mg/kg, s.c., −0.5 h; *n* = 5 per dose) was investigated. Two-way analysis of variance (ANOVA) followed by Bonferroni's posttests was performed to establish the statistical significance between test groups.

### Conditioned avoidance behavior in rats

The apparatus has been previously described (Megens et al. 2014). Rats were trained to avoid an electric shock during 5 sessions at 15-min time intervals during a 1-h period. In each training session, the rat was placed on the nonelectrified grid floor and the grid was electrified 10 sec later for not more than 30 sec, if the rat did not jump out of the box. Only those rats showing a correct conditioned avoidance response, that is, jumping before shock (latency <10 sec) in the last 3 training sessions were included in the experiment. Selected animals were dosed with the test compounds (JNJ-4011813, JNJ-42153605, LY404039, ritanserin), subcutaneously (s.c.), immediately after the last training session and were tested at 60, 90, and 120 min thereafter for avoidance behavior. Latency to avoidance (responding within the 10-sec interval before the grid is electrified) or escape response (responding after the grid has been electrified; cutoff time: 10 sec) was recorded manually. The median avoidance response and the maximum escape response obtained over the three experimental sessions per rat were used for analysis. A median avoidance response >8 sec occurred in only 1.8% of vehicle-pretreated control rats (*n* > 400) and was selected as an all-or-none criterion for drug-induced inhibition of avoidance. A maximum escape response >9 sec over the three trials never occurred in these control rats and was adopted as an all-or-none criterion for inhibition of escape. ED_50_ values and corresponding 95% confidence intervals were determined for both effects as described below.

### DOM-induced head twitches in rats

The DOM (0.63 mg/kg, i.v.)-induced head twitches were counted over the first 15 min after DOM injection in rats pretreated s.c. 1 h (2 h for JNJ-40411813) earlier with test compounds or vehicle. The DOM challenge dose was selected as the lowest dose reliably inducing >5 head twitches in 95% of vehicle-pretreated control rats. Criterion for drug-induced inhibition: <5 head twitches (false positives in controls: 8.6%; *n* = 486). ED_50_s and corresponding 95% confidence intervals were determined as described below.

### Determination of ED_50_ values

The ED_50_ values (the “median effective dose” that produces a quantal effect [all or none] in 50% of the animals tested with that dose) and corresponding 95% confidence intervals were determined for the above tests. The all-or-none criteria were defined by analyzing a frequency distribution of a series of historical control data obtained in vehicle-treated animals, aiming for <5% responders in this control population. The fraction of animals responding to a criterion was determined per dose level. All doses in the dose range relevant to the pharmacological effect, that is, from the highest dose inducing the minimum number of responders (usually 0%) to the lowest dose inducing the maximum number of responders (usually 100%), were tested in at least 5 animals. The doses producing 50% responders to criterion were determined according to the modified Spearman–Kaerber estimate, using theoretical probabilities instead of empirical ones (Tsutakawa 1982). This modification allowed the determination of the ED_50_ and its confidence interval as a function of the slope of the log dose–response curve. This method has been used for several publications (e.g., Megens et al. 2014) and an internal report on the method is available upon request (Lewi et al. 1977).

### Memantine-induced brain activation in mice

Mice (*n* = 10–30/group) were treated with JNJ-40411813 (10 and 40 mg/kg, s.c.), JNJ-42153605 (2.5 and 10 mg/kg s.c.), ritanserin (0.16 and 0.63 mg/kg, s.c.) or vehicle, followed by treatment with memantine (20 mg/kg, s.c.) 30 min later and with [^14^C]2-deoxyglucose (^14^C2DG; 0.16 *μ*Ci/g, i.p.) 45 min later. Forty-five min after ^14^C2DG administration, animals were sacrificed by decapitation, brains were isolated and frozen in cold 2-methylbutane (−30°C, on dry ice), and stored at −20°C until sectioned. Serial coronal sections (20 *μ*m thick) were collected 2.10, 1.10, and −1.94 mm from bregma on glass slides using a cryostat (Leica CM 3050; van Hopplynus Instruments, Brussels, Belgium), and dried rapidly on a hot plate at 60°C. Brain sections were then exposed to Biomax films (Kodak, Perkin Elmer, Perkin, Elmer, Waltham, MA, UK), together with a precalibrated [^14^C]standard (ARC, Saint Louis, MO, USA). The autoradiographic films were developed after 4 days of exposure. Local tissue [^14^C] concentration (nCi/mg tissue equivalent) in each region of interest was determined using a computer-based image analysis system (MCID Basic 7.0).

Analysis was limited to the molecular layer of the hippocampus, based on previously published reports (Duncan et al. 1998a,b; Miyamoto et al. 2000) and on our own results showing the most robust activation by memantine in this area (Dedeurwaerdere et al. 2011). Based on the previously reported dose–response data (Dedeurwaerdere et al. 2011), a challenge dose of memantine of 20 mg/kg was selected for reversal studies. The hippocampal molecular layer was outlined manually as per the stereotaxic atlas of (Paxinos and Franklin 2001), and the bilateral density readings on three consecutive brain sections were recorded, averaged and used for further analysis.

The data were analyzed using a two way ANOVA, followed by a Bonferroni posthoc test to compare the brain activity in vehicle-treated animals with and without memantine challenge; and to compare the memantine response versus reversal of memantine response by the test compound.

### Acoustic startle response in mice

Mice were injected with JNJ-40411813 (2.5, 5, 10, 20, or 40 mg/kg, s.c.), JNJ-42153605 (2.5, 5 or 10 mg/kg, s.c.) or LY404039 (0.63, 2.5, 10 mg/kg, s.c.) 30 min before testing and were placed in an individual enclosure, which was placed in a startle reflex chamber (San Diego Instruments, San Diego, CA, US) 5 min prior to the start of the session. A continuous background noise of 65 dB was maintained. The animals were exposed to a random mix of 14 blocks of 7 sound pulses (40 msec) of different intensity (no pulse [65 dB background noise], 70, 80, 90, 100, 110, and 120 dB). The amplitude of the startle response to each sound intensity level was recorded using a piezoelectric sensor attached to the bottom of the enclosure. The mean startle response to each of the 14 pulses of a given strength was calculated. The data were analyzed using a two-way repeated measures ANOVA with pulse intensity as within- and treatment as between-subject factors. Appropriate decompositions with contrast and multiple comparisons with vehicle were used to detect drug effects versus vehicle.

### Rotarod test in rat

Rats were trained on the rotarod at constant speed on day 1 in three sessions of 5 min each (16, 20, 24 rpm, respectively), followed by a session at linearly increasing speed from 6 to 30 rpm over 4 min. On day 2, animals were tested 4 times (30 min intervals) at incremental speed (6–30 rpm over 4 min); compounds were administered immediately after the first session. Animals performing less than 100 sec on the rotarod in the session prior to drug-administration were excluded from the study, and the remaining animals were randomized to one of the following treatment groups: JNJ-40411813 (2.5–40 mg/kg p.o.), JNJ-42153605 (5–20 mg/kg p.o.) or LY404039 (2.5–40 mg/kg p.o.), or the respective vehicle. The latency time on the rotarod (time at which the animals fell of the rod) was recorded. Animals that completed the entire session were assigned the maximum latency time of 240 sec. Latency times for each animal were normalized to the pretreatment value and expressed as a percentage thereof for statistical analysis by repeated measures ANOVA.

## Results

### Locomotor activity in mice

**Figure 2:**
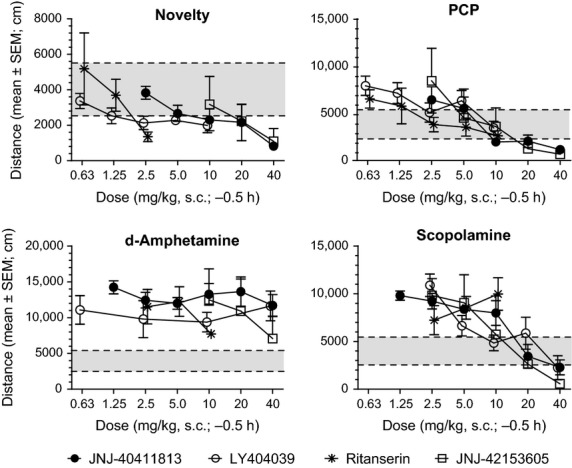
Effects of JNJ-40411813, JNJ-42153605, LY404039, and ritanserin on spontaneous locomotion and on hyperlocomotion induced by PCP, d-amphetamine or scopolamine. The gray area represents the control range for locomotion observed in nonchallenged rats. The broken horizontal lines reflect the critical levels for inhibition of hyperlocomotion (<5500 cm) and for blockade of locomotion (<2500 cm).

JNJ-40411813, JNJ-42153605, and LY404039 dose dependently inhibited spontaneous locomotion and PCP- and scopolamine-induced hyperlocomotion but not d-amphetamine-induced hyperlocomotion (Fig.2; Table2). Progressively higher doses further reduced the PCP- and scopolamine-induced locomotion to values (<2500 cm) below the spontaneous activity level measured in mice not challenged with stimulants. Also ritanserin inhibited spontaneous locomotion and PCP-induced hyperlocomotion but not d-amphetamine- or scopolamine-induced hyperlocomotion.

**Table 2:** ED_50_s (and 95% confidence limits; mg/kg, s.c.) or lowest active dose (mg/kg, s.c.) of the test compounds for the listed effects in mice and rats at the indicated time interval after s.c. injection.

		ED_50_s (and 95% confidence limits; mg/kg, s.c.) or lowest active dose (mg/kg, s.c.)			
Effects	Time (h)	JNJ-40411813	JNJ-42153605	LY404039	Ritanserin
Mice					
Spontaneous locomotion, blockade	0.5–1	7.1 (4.4–11.5)	15.2 (10.8–21.3)	1.78 (0.98–3.2)	0.67 (0.39–1.16)
PCP hyperlocomotion, inhibition	0.5–1	5.4 (3.6–8.0)	5.4 (3.8–7.5)	3.3 (2.17–5.0)	2.35 (1.45–3.8)
PCP hyperlocomotion, blockade	0.5–1	10.8 (6.3–18.5)	11.4 (8.2–16.1)	10 (–)	>10
Scopolamine hyperlocomotion, inhibition	0.5–1	14.2 (9.5–21)	7.1 (4.7–10.6)	10.8 (6.3–18)	>10
Scopolamine hyperlocomotion, blockade	0.5–1	21.5 (14.4–32)	8.1 (5.4–12.2)	32 (24.0–44)	>10
d-Amphetamine hyperlocomotion, inhibition	0.5–1	>40	≥40	>10	>10
d-Amphetamine-hyperlocomotion, blockade	0.5–1	>40	≥40	>10	>10
Spontaneous 2-DG uptake, inhibition	0.75–1.5	40	10	>10	>0.63
Memantine 2-DG uptake, inhibition	0.75–1.5	40	10	10^2^	0.63
Enhancement of acoustic startle, inhibition	0.5	>40	>10	2.5	Not tested
Rats					
Avoidance behavior, inhibition	1–2	24.7 (16.5–37)	2.36 (1.37–4.0)^3^	2.04 (1.36–3.0)^3^	≥20
Escape behavior, inhibition	1–2	>40	21.5 (14.4–32)^3^	8.1 (5.4–12.2)^3^	>20
DOM head twitches, inhibition	1	4.7 (3.1–7.0)^1^	10.7 (7.9–14.6)	1.77 (1.44–2.18)	0.223 (0.130–0.38)
Rotarod performance, inhibition	0.5–1.5	>40 (p.o.)	>20 (p.o.)	2.5 (p.o.)	Not tested

1 ED_50_ obtained 2 h after s.c. dosing.

2 Previously reported (Dedeurwaerdere et al. 2011).

3 Previously reported (Megens et al. 2014).

**Figure 3:**
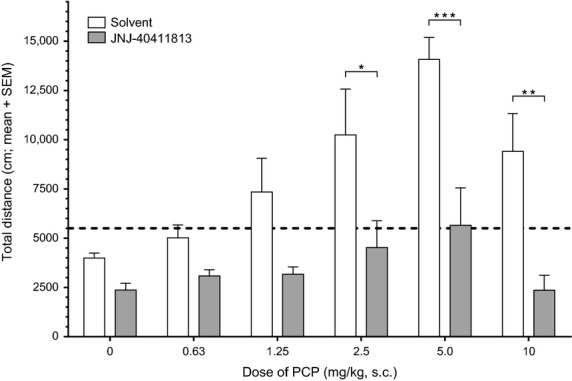
Effect of JNJ-40411813 (10 mg/kg, s.c., −0.5 h) versus vehicle (10 mL/kg, s.c., −0.5 h) on the dose–response curve of PCP (0, 0.63, 1.25, 2.5, 5.0, or 10 mg/kg, s.c.; *n* = 5 per test group) for stimulation of locomotion in mice. The bars represent mean (+SEM) values for distance traveled. The dotted horizontal line indicates the critical level adopted for induction of hyperlocomotion (>5500 cm). *P < 0.05, **P < 0.01, ***P < 0.001 (Bonferroni posttests)

In a separate study, the effect of cotreatment with JNJ-40411813 (10 mg/kg, s.c., 30 min) versus vehicle on the dose–response curve of PCP (0, 0.63, 1.25, 2.5, 5.0, 10 mg/kg, s.c., 30 min, *n* = 5 per dose) for stimulation of locomotion was investigated. In mice cotreated with vehicle, PCP increased total distance, with a bell-shaped dose–response relation (Fig.3). In mice cotreated with JNJ-40411813, the effect of PCP was reduced relative to that measured after vehicle cotreatment. Although the effect was statistically significant only at the PCP doses of 2.5, 5.0, and 10 mg/kg, JNJ-40411813 apparently reduced locomotion to a similar extent at all doses of PCP including the vehicle group, without shifting the dose–response curve (Fig.3).

### Memantine-induced brain activation in mice

**Figure 4:**
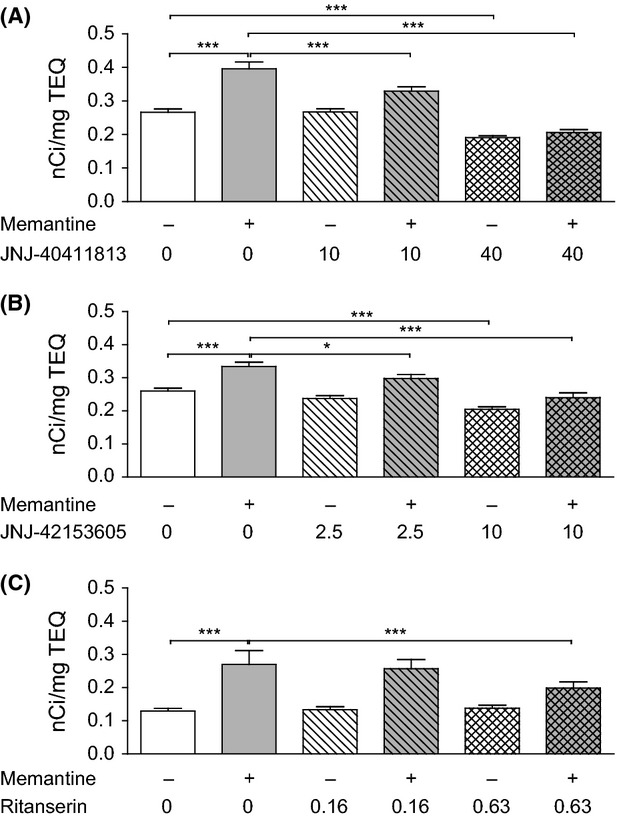
Effect of JNJ-40411813, JNJ-42153605, and ritanserin on memantine (20 mg/kg, s.c.)-induced brain activation in mice. The figure shows [^14^C]2DG uptake in the hippocampus across treatment groups. Each compound data set was analyzed using a mixed two-way ANOVA analysis, followed by post hoc tests (one-sided for comparison of control vs. memantine response and memantine response vs. reversal by the compound, two-sided for compound effect without memantine challenge). Data are shown as mean ± SEM, (*P < 0.05; ***P <* *0.001).

Memantine (20 mg/kg, s.c.) significantly increased brain activation (brain glucose metabolism) in the hippocampus (Fig.4). In this region, JNJ-40411813 at 10 mg/kg (s.c.) significantly reduced the effect of memantine on [^14^C]2DG uptake; the effects of combined JNJ-40411813/memantine treatment were not significantly different from control at a dose of JNJ-40411813 (i.e., 40 mg/kg s.c.) that decreased brain activation when given alone (Fig.4A). In line with previously reported data with LY404039 (Dedeurwaerdere et al. 2011), the selective mGlu2 PAM JNJ-42153605 inhibited memantine-induced 2DG uptake (Fig.4B). Interestingly, ritanserin (at 0.63 mg/kg s.c.) also blocked the effect of memantine (Fig.4C).

### Conditioned avoidance response in rats

**Figure 5:**
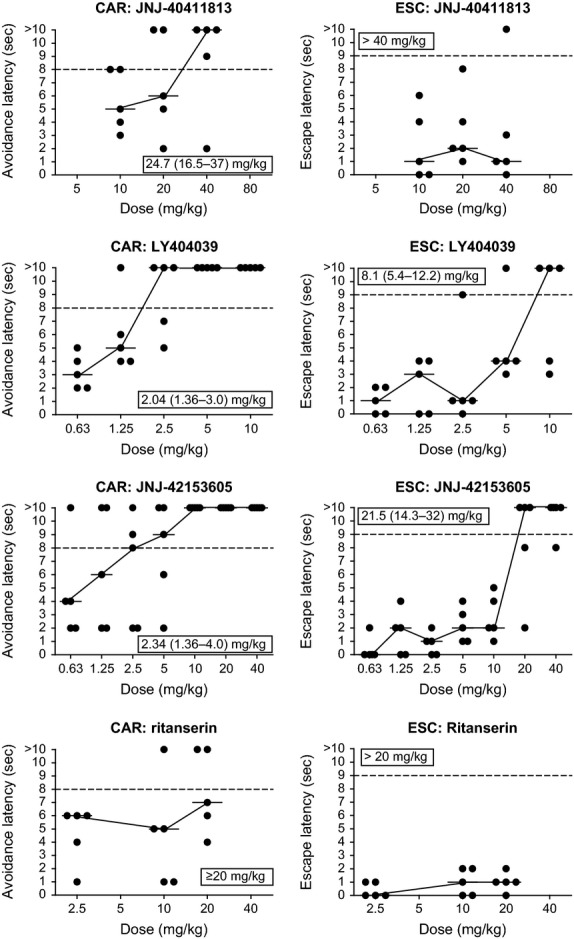
Effect of JNJ-40411813 on the conditioned avoidance (CAR) and escape (ESC) behavior in rats: Comparison with the mGlu2/3 agonist LY404039, the mGlu2 PAM JNJ-42153605, and the 5HT_2A_ antagonist ritanserin. Shown are individual and median response latencies (circles and stripes, respectively) per dose level. The horizontal dotted lines represent the critical levels for inhibition of avoidance (latency > 8 sec) and blockade of escape behavior (>9 sec). ED_50_s (and 95% confidence intervals) are given in the inserts. Results obtained 1–2 h after dosing.

JNJ-40411813 inhibited conditioned avoidance responding but did not affect escape behavior within the dose range tested (Fig.5; Table2). As reported earlier (Megens et al. 2014), LY404039 and JNJ-42153605 also inhibited conditioned avoidance responding. Ritanserin was inactive up to 20 mg/kg s.c.

### DOM-induced head twitches in rats

Like ritanserin, JNJ-40411813, JNJ-42153605 and LY404039 dose dependently inhibited DOM-induced head twitches. The obtained ED_50_s have been listed in Table2.

### Acoustic startle response in mice

**Figure 6:**
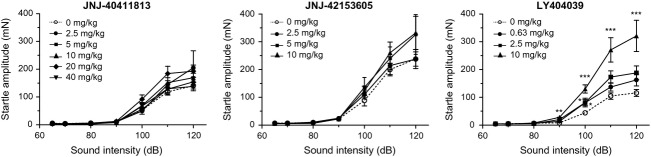
Effect of JNJ-40411813, JNJ-42153605, and LY404039 on acoustic startle response amplitude in mice. Data are shown as mean ± SEM (*n* = 10–20/group, doses in mg/kg, s.c., 30 min). Repeated measures-ANOVA: *P < 0.05, **P < 0.01, ***P < 0.001 versus vehicle.

Vehicle-treated mice responded with a stimulus-dependent increase in startle response amplitude from 100 dB onward (Fig.6). The mGlu2 receptor PAMs JNJ-40411813 (up to 40 mg/kg s.c.) or JNJ-42153605 (up to 10 mg/kg s.c.) did not statistically significantly affect startle response amplitude. LY404039 (s.c.) did not change startle response amplitude at 0.63 mg/kg but produced a pronounced dose-dependent and statistically significant (*P* < 0.05, *n* = 20/group) increase from 90 dB onward at 2.5 and 10 mg/kg; the increase in startle response amplitude was up to 2.5-fold at 120 dB for the highest dose tested.

### Rotarod test in rat

**Figure 7:**
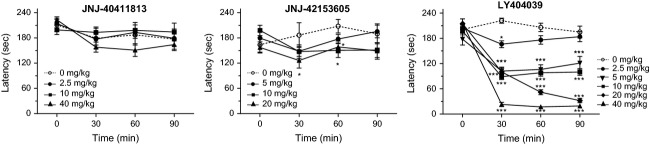
Effect of JNJ-40411813, JNJ-42153605, and LY404039 on latency of rats to fall off the rotarod at incremental speed. Doses are expressed as mg/kg, p.o.; data are shown as mean ± SEM for *n* = 7–20. Repeated measures ANOVA: *P < 0.05, ***P < 0.001 versus vehicle.

Rats treated with JNJ-40411813 showed no difference in rotarod performance versus vehicle treatment up to 40 mg/kg (p.o.). Treatment with 20 mg/kg p.o. JNJ-42153605 resulted in a small but statistically significant effect at 30 and 60 min after administration, full recovery was observed at 90 min. However, LY404039 significantly reduced performance at 30 min after 2.5 mg/kg p.o. and at every time point from 5 mg/kg p.o. onward (Fig.7).

## Discussion

mGlu2 receptor PAMs potentiate the activity of the endogenous agonist glutamate, which may offer treatment advantages over direct orthosteric agonists. We compared the effects of JNJ-40411813, a novel mGlu2 receptor PAM with associated 5HT_2A_ antagonism in rats (due to the generation of a rodent-specific metabolite), with the mGlu2-selective PAM JNJ-42153605, the mGlu2/3 orthosteric agonist LY404039, and the 5HT_2A_ antagonist ritanserin in animal models for antipsychotic activity and side effects, in an attempt to differentiate between compounds and mechanisms of action. The combined mGlu2 PAM/5HT_2A_ antagonistic activity of JNJ-40411813 is of particular interest since both serotonin 5HT_2A_ and mGlu2 receptors have been implicated in the pathophysiology of schizophrenia, as well as in the mechanism of action of hallucinogenics (Aghajanian 2009; Gonzalez-Maeso and Sealfon 2009). Moreover, the 5HT_2A_ and the mGlu2 receptor form a heteromeric complex through which serotonin and glutamate ligands modulate the pattern of G protein coupling (Fribourg et al. 2011).

Similar to LY404039, the mGlu2 PAMs JNJ-40411813 and JNJ-42153605 inhibited spontaneous locomotion and PCP- and scopolamine-induced hyperlocomotion but not d-amphetamine-induced hyperlocomotion in mice. These data indicate that mGlu2 PAMs and agonists differ from currently available antipsychotics that act by blocking dopamine D_2_ receptors, and which inhibit spontaneous locomotion as well as PCP- and d-amphetamine-induced hyperlocomotion. Ritanserin, a selective 5HT_2A_ antagonist inhibited spontaneous locomotion and PCP-induced hyperlocomotion but not d-amphetamine or scopolamine-induced hyperlocomotion. The scopolamine-challenge model specifically picked up mGlu2-mediated activity, confirming that among the complex mechanisms of action of scopolamine, increasing extracellular glutamate concentrations in the prefrontal cortex (Voleti et al. 2013) could account for the effects observed with mGlu2 modulators. The observed effects of mGlu2 ligands on PCP-induced behavioral changes are consistent with previous reports, as is the effect of 5HT_2A_ antagonism on PCP-induced hyperactivity (Gleason and Shannon 1997). As shown for JNJ-40411813, the inhibition of PCP-induced hyperlocomotion resulted from a decrease of effect size at all doses of PCP and not from a shift of the dose–response curve. The low degree or virtual absence of specificity for inhibition of PCP- or scopolamine-induced hyperlocomotion versus inhibition of spontaneous locomotion suggests overall behavioral suppression to be responsible. On the other hand, the failure of the compounds to inhibit d-amphetamine-induced hyperlocomotion argues against nonspecific behavioral suppression.

JNJ-40411813, JNJ-42153605, and LY404039 also inhibited conditioned avoidance behavior in rats, another model considered predictive for evaluation of antipsychotic activity (Schlumberger et al. 2010; Wadenberg 2010). While JNJ-42153605 and LY404039 inhibited escape behavior, JNJ-40411813 was ineffective, presumably due to the small dose range tested (dosing was limited by poor solubility). JNJ-40411813, JNJ-42153605, and LY404039 inhibited conditioned avoidance responding, whereas ritanserin – even when tested at doses that correspond to maximal 5HT_2A_ receptor occupancy (>90% occupancy at 2.5 mg/kg s.c.; data not shown) or that are 100-fold the dose active against DOM-induced head twitches- failed to do so, attesting to the mGlu2-specificity of the observed effects, and confirming earlier findings (Megens et al. 2014). The interpretation of the effects on conditioned avoidance highly depends on the specificity of these effects relative to effects on escape behavior and motor activity. The specificity margins obtained in our hands are rather small, also for neuroleptics (Megens et al. 2014). Although inhibition of conditioned avoidance by neuroleptics appears to be highly correlated with antidopaminergic activity and with clinical potency for this class of compounds, the notion of selective blockade of conditioned avoidance as a specific predictor of antipsychotic activity is not generally valid or at best dependent on the procedures employed (Castagne et al. 2009).

Autoradiographic assessment of radiolabeled 2DG uptake is commonly used to investigate changes in brain activity. The NMDA receptor antagonist memantine robustly stimulates brain glucose metabolism in mice, similarly to ketamine (Duncan et al. 1998b). We previously showed that the increase in 2DG uptake induced by memantine can be reversed by the atypical antipsychotic clozapine, but not by the typical antipsychotic haloperidol, suggesting that reversal of memantine-induced increases in brain glucose metabolism depends on receptors other than the dopamine D_2_ receptor, which is classically associated with antipsychotic activity (Dedeurwaerdere et al. 2011). In this study, JNJ-40411813 reversed memantine-induced brain activation in a dose-dependent manner with complete reversal at 40 mg/kg. Importantly, both the mGlu2-selective PAM JNJ-42153605 and ritanserin prevented the rise in 2DG levels, suggesting that mGlu2 and 5HT_2A_ receptors may be directly involved in the observed effects. Alternatively, as mGlu2/3 receptors show an overlapping cellular distribution with 5HT_2A_ receptors in brain cortex and mGlu2 activators have been shown to suppress serotonin-evoked postsynaptic currents (Benneyworth et al. 2007; Rorick-Kehn et al. 2007a), our data may imply that the 5HT_2A_ receptor is the key mediator for the effects observed with mGlu2 modulators, as well as atypical antipsychotics-like clozapine.

In line with earlier findings that mGlu2/3 receptor activation blocks the in vivo behavioral effects induced by 5HT_2A_ receptor agonists-like DOM (Gewirtz and Marek 2000; Klodzinska et al. 2002), in the present study, all three mGlu2 receptor ligands inhibited DOM-induced head twitches in rats. While mGlu2-5HT_2A_ functional (indirect) antagonism has been well established, it is unclear whether or to which extent indirect inhibition of 5HT_2A_ receptor activity may contribute to the antipsychotic potential of mGlu2 ligands.

The mGlu2 receptor PAMs JNJ-40411813 and JNJ-42153605 did not alter the acoustic startle response, while LY404039 showed a significant and dose-dependent rise from 90 dB onwards. The enhanced response with the agonist may reflect exaggerated processing as a result of continuous mGlu2 or mGlu3 receptor activation and corresponding potentiated glutamatergic signaling, or speculatively the mGlu3 agonist activity of LY404039 may be responsible for the observed effects. Anyhow, the LY404039-induced acoustic startle enhancement is antagonized by the orthosteric mGlu2/3 antagonist LY341495 (Megens et al. 2014).

LY404039 as well as JNJ-40411813 and JNJ-42153605 reduced spontaneous locomotion. In the rotarod model, JNJ-40411813, up to a dose of 40 mg/kg p.o., did not alter the motor function, while JNJ-42153605 only showed a minor and transient effect. However, LY404039 significantly reduced rotarod performance from 5 mg/kg p.o onward. As Rorick-Kehn et al. (2007b) reported no impairment by LY404039, it should be noted that we used a more stringent protocol with an incremental rotation speed from 6 to 30 rpm. While both the increase in acoustic startle response and reduction in rotarod performance indicate that mGlu2/3 receptor agonists can be differentiated from mGlu2 PAMs based on their preclinical secondary activity profile, it is unclear to what extent this translates to clinically relevant differences. LY2140023, the prodrug of LY404039, was tested in several large clinical trials and, aside from convulsions in some occasions, it was well tolerated (Kinon et al. 2011; Stauffer et al. 2013); both in healthy volunteers and patient populations (schizophrenia, anxious depression), also JNJ-40411813 was well tolerated and showed no serious treatment-emergent adverse events (Kent et al. 2013).

Collectively, our studies suggest that mGlu2 activation alone is sufficient for effects on PCP- and scopolamine-induced hyperlocomotion in mice, memantine-induced 2DG uptake in mice, conditioned avoidance responding and DOM-induced head twitches in rats, since both JNJ-40411813, JNJ-42153605 as well as LY404039 are effective in these models. As ritanserin shows activity in some of these models (PCP-induced hyperlocomotion, memantine-induced brain activation and DOM-induced head twitches), the question remains, however, whether 5HT_2A_ antagonism contributes to some of the effects observed with JNJ-40411813. Relatively high doses of JNJ-42153605 were needed to inhibit DOM-induced head twitches relative to its ED_50_ for mGlu2 occupancy (ED_50_ DOM model = 10.7 mg/kg s.c. vs. ED_50_ occupancy = 1.1 mg/kg p.o., data not shown, te Riele et al., 2014), while for JNJ-40411813, 50% occupancy is reached at a dose between 2.5 and 10 mg/kg s.c. (Lavreysen et al submitted[Bibr b47]) and the ED_50_ for inhibiting DOM effects is “only” 4.7 mg/kg s.c. Hence, while 5HT_2A_ antagonism may partly mediate JNJ-40411813's effects against DOM, these effects were likely mediated via the in vivo generation of a metabolite with potent 5HT_2A_ antagonistic activity.

Similarly, as relatively high mGlu2 receptor occupancy is needed to drive the PCP antagonistic effect of the more potent and selective mGlu2 PAM JNJ-42153605 (ED_50_ PCP model = 5.4 mg/kg s.c. vs. ED_50_ mGlu2 occupancy = 1.1 mg/kg p.o.; data not shown; te Riele et al., 2014), it cannot be excluded that both mGlu2 and 5HT_2A_ receptors contribute to the activity of JNJ-40411813 in the PCP model (ED_50_ for activity ≤ ED_50_ occupancy). It is of note that caution should be taken when extrapolating doses required for activity to doses for occupancy because of potential species differences (occupancy is tested in rats while the locomotion assays were all performed in mice), and the fact that different levels of occupancy may be needed for activity of PAMs based on different degrees of positive cooperativity.

The top-line data of a recent clinical study in patients with anxious depression did not support further development of JNJ-40411813 for this indication (Kent et al. 2014). However, data from an exploratory schizophrenia trial indicate that add-on treatment with JNJ-40411813 may provide benefit to patients with prominent negative symptoms of schizophrenia (De Boer et al. 2013; Kent et al. 2013). JNJ-40411813 also attenuated ketamine-induced increases in Brief Psychiatric Rating Scale (BPRS) scores, mostly via an effect on negative symptoms (De Boer et al. 2013). Importantly, though contribution of 5HT_2A_ antagonism to the effects of JNJ-40411813 cannot be excluded in rodents, it does not play a role in man. Circulating levels of the JNJ-40411813 metabolite with high 5HT_2A_ affinity are negligible in man, and only 10–25% 5HT_2A_ occupancy ([^11^C]MDL 100,907 PET) was measured at clinically relevant doses (Hoeben et al. 2013). All clinical effects of JNJ-40411813 can therefore be attributed to its mGlu2 PAM effect. While the different in vivo pharmacological profile of JNJ-40411813 (i.e., both mGlu2 receptor PAM and 5HT_2A_ receptor antagonism in rodents vs. only mGlu2 receptor PAM in man) makes direct back-translation less straightforward, robust in vivo pharmacodynamic effects were observed in several animal models, very similar to the effects reported for other mGlu2 ligands, including LY404039. Without a direct way to assess mGlu2 receptor target engagement in man, it does remain an open question whether sufficiently high doses of JNJ-40411813 and corresponding levels of mGlu2 receptor occupancy were reached to elicit the antipsychotic effects that may be anticipated from the preclinical models and/or whether these models really predict clinical efficacy for novel, nondopaminergic mechanisms of action. In this respect, it is important to note that relatively high doses of JNJ-40411813 were required to achieve effects in some models such as conditioned avoidance responding in rats, corresponding to plasma levels that were not reached in the clinic (unpublished observations).

In conclusion, our studies show that mGlu2 modulation leads to robust in vivo pharmacodynamic effects; there was no apparent difference in activity profile between JNJ-40411813 and JNJ-42153605, indicating that while additional 5HT_2A_ antagonism may contribute to certain in vivo effects, it does not differentiate these mGlu2 receptor PAMs based on their in vivo activity profile. Only scopolamine-induced hyperlocomotion in mice and conditioned avoidance responding in rats seem to be affected by mGlu2 activation or modulation and not via 5HT_2A_ modulation. The mGlu2 receptor PAMs mimicked the in vivo pharmacodynamic effects observed with the agonist LY404039 except for impairment of rotarod performance and enhancement of acoustic startle, suggesting that these mGlu2 PAMs produce a primary activity profile similar to that of mGlu2/3 receptor agonists while they can be differentiated based on their secondary activity profile.

## Abbreviations

[^14^C] 2DG: [^14^C]2-deoxyglucose
5HT_2A_: 5-Hydroxytryptamine
ANOVA: analysis of variance
DOM: 2,5-dimethoxy-4-methylamphetamine
JNJ-40411813: 1-butyl-3-chloro-4-(4-phenylpiperidin-1-yl)pyridin-2(1H)-one
JNJ-42153605: 3-(cyclopropylmethyl)-7-(4-phenylpiperidin-1-yl)-8-(trifluoromethyl)[1,2,4]triazolo[4,3-a]pyridine
LED: light-emitting diode
LY341495: 2-(2-Carboxycyclopropyl)-3-(9H-xanthen-9-yl)-D-alanine
LY354740: (1S,2S,5R,6S)-(+)-2-aminobicyclo[3.1.0]hexane-2,6-dicarboxylic acid
LY379268: (-)-2-oxa-4-aminobicyclo[3.1.0]hexane-4,6-dicarboxylic acid
LY379268: 4-amino-2-oxabicyclo[3.1.0]hexane-4,6-dicarboxylic acid
LY404039: 4-amino-2-thiabicyclo[3.1.0]hexane-4,6-dicarboxylic acid 2,2-dioxide
mGlu: metabotropic glutamate
PAM: positive allosteric modulator
PCP: phencyclidine or 1-(1-phenylcyclohexyl)piperidine
PVC: polyvinyl chloride
SD: Standard deviation
SEM: Standard error of mean
